# A component of the Sec61 ER protein transporting pore is required for plant susceptibility to powdery mildew

**DOI:** 10.3389/fpls.2013.00127

**Published:** 2013-05-16

**Authors:** Wen-Jing Zhang, Susanne Hanisch, Mark Kwaaitaal, Carsten Pedersen, Hans Thordal-Christensen

**Affiliations:** Section for Plant and Soil Science, Department of Plant and Environmental Sciences, Faculty of Science, University of CopenhagenFrederiksberg, Denmark

**Keywords:** powdery mildew, haustorium, extrahaustorial membrane (EHM), endoplasmic reticulum-associated degradation (ERAD), Sec61 complex, susceptibility factor

## Abstract

Biotrophic pathogens, like the powdery mildew fungi, require living plant cells for their growth and reproduction. During infection, a specialized structure called the haustorium is formed by the fungus. The haustorium is surrounded by a plant cell-derived extrahaustorial membrane (EHM). Over the EHM, the fungus obtains nutrients from and secretes effector proteins into the plant cell. In the plant cell these effectors interfere with cellular processes such as pathogen defense and membrane trafficking. However, the mechanisms behind effector delivery are largely unknown. This paper provides a model for and new insights into a putative transfer mechanism of effectors into the plant cell. We show that silencing of the barley Sec61βa transcript results in decreased susceptibility to the powdery mildew fungus. *Hv*Sec61βa is a component of both the endoplasmic reticulum (ER) translocon and retrotranslocon pores, the latter being part of the ER-associated protein degradation machinery. We provide support for a model suggesting that the retrotranslocon function of *Hv*Sec61βa is required for successful powdery mildew fungal infection. *Hv*Sec61βa-GFP and a luminal ER marker were co-localized to the ER, which was found to be in close proximity to the EHM around the haustorial body, but not the haustorial fingers. This differential EHM proximity suggests that the ER, including *Hv*Sec61βa, may be actively recruited by the haustorium, potentially to provide efficient effector transfer to the cytosol. Effector transport across this EHM-ER interface may occur by a vesicle-mediated process, while the Sec61 retrotranslocon pore potentially provides an escape route for these proteins to reach the cytosol.

## INTRODUCTION

Many filamentous plant pathogenic fungi and oomycetes rely on placing a feeding structure, a so-called haustorium inside host cells in order to exploit host resources and to transfer effector proteins to the host cytosol. By unknown mechanisms, these pathogens trigger the host cells to generate an extrahaustorial membrane (EHM), which allows the host cells to stay alive despite the severe haustorial invasions (Gan et al., 2012). In between the haustorium and the EHM, a sealed compartment, called the extrahaustorial matrix (EHMx) is present. Many of these pathogens, such as powdery mildew fungi, have genetically lost certain general life-sustaining processes during their evolution (Spanu et al., 2010). This prevents them from living on dead biological material, making them strict biotrophs. In the meantime, they secrete hundreds of effectors from the haustoria, mediated by signal peptides (SPs) and default secretion. Many of these effectors are transferred to the host cytosol, where they play important roles in pathogenicity by assisting in nutrient acquisition, suppression of defense and reprogramming cellular processes (Bozkurt et al., 2012; Pedersen et al., 2012; Saunders et al., 2012). An inherent problem, which is poorly understood, concerns how the effectors escape the EHM-delimited haustorial compartment to access the plant cytosol. This requires a mechanism to cross membranes, such as a protein transmitting pore. Essentially, the only currently established element of this process is the RxLR-dEER motif, located a few amino acids downstream of the SP cleavage site in many oomycete effectors. By an unknown process, this motif guides the effectors to be transported across membranes and allows them to enter the host cytosol (Whisson et al., 2007).

The endoplasmic reticulum (ER) is a major organelle in eukaryotic cells, which forms an extended network, functioning in, e.g., protein processing and sorting. Voegele et al. (2009) have previously suggested that the ER plays a role in transfer of effector to the plant cytosol. In the ER, proper folding and modification of proteins is assisted and validated by the ER quality control (ER-QC) machinery. If proteins finally fail the quality check, they are recognized by the ER-associated protein degradation (ERAD) machinery and retrotranslocated into the cytosol to be degraded by proteasomes (Nakatsukasa and Brodsky, 2008). Effectors may exploit this retrotranslocon pore in order to get access to the plant cytosol. Different multicomponent retrotranslocon pores have been described in yeast and mammals, in which, e.g., Derlin, Hrd, and Sec61 proteins are major elements (Kawaguchi and Ng, 2007; Nakatsukasa and Brodsky, 2008). The ERAD substrates are ubiquitinated during the retrotranslocation process by retrotranslocon-associated ubiquitin ligases, and this targets them for proteasomal degradation as soon as they reach the cytosol (Carvalho et al., 2006, 2010). The Sec61 pore can translocate proteins bi-directionally, and it is primarily known as the translocon pore, mediating the process of SP-dependent protein translocation into the ER. The Sec61 pore is a doughnut-shaped heterotrimeric complex, consisting of the subunits, Sec61α, Sec61β, and Sec61γ. SP and Sec61-dependent translocation into the ER can occur either co- or post-translationally (Zimmermann et al., 2011). The ERAD pathway has in several cases been shown to be recruited by opportunistic pathogens for transfer of polypeptides into the host cell cytosol. For example, cholera toxin, shiga toxin, and *Pseudomonas aeruginosa* exotoxin enter the cytosol through retrotranslocon pores, but escape from ubiquitination and proteasome-mediated degradation (Rodighiero et al., 2002; Blanke, 2006). Retrotranslocation of cholera toxin occurs through the Sec61 retrotranslocon pore, and depletion of the Sec61 complex prevented the retrotranslocation of this toxin into the cytosol (Schmitz et al., 2000; Teter et al., 2002).

Here we aimed at studying the role of the Sec61 pore in plant susceptibility to the powdery mildew fungus. Barley (*Hordeum vulgare*) has two Sec61α, two Sec61β, and one Sec61γ protein^1^ (Deng et al., 2007; Mayer et al., 2012), and to unravel the role of the pore, we made use of the fact that the Sec61β component is essential for retrotranslocon activity for various substrates, but less important for translocon activity under non-stressed conditions (Finke et al., 1996; Van den Berg et al., 2004; Liao and Carpenter, 2007; Kelkar and Dobberstein, 2009; Zhao and Jantti, 2009; Wang et al., 2010; Guerriero and Brodsky, 2012). We show that silencing of* HvSec61βa *reduced the susceptibility of barley epidermal cells to the powdery mildew fungus (*Blumeria graminis *f.sp. *hordei*, *Bgh*). In addition, the *Hv*Sec61βa-GFP-labeled ER network is differentially associated to the body, and not the fingers of the powdery mildew fungal haustorium. To explain the role of the Sec61βa in pathogenicity, we propose a model in which the fungus actively recruits the ER in order to exploit the Sec61 pore for pathogenicity.

## MATERIALS AND METHODS

### PLANTS AND FUNGI

Barley (*Hordeum vulgare*) cv. Golden Promise plants were used for transient transformation and subsequent studies with and without powdery mildew fungal inoculation. The barley powdery mildew fungus (*Blumeria graminis *f.sp. *hordei*, *Bgh*), isolate DH14, was maintained on susceptible barley, cv. Golden Promise, grown at 20°C, 16 h light (150 μE/sm^2^)/8 h dark, by weekly inoculum transfer. These growth conditions were used throughout the studies.

### CLONING

To generate a gene-specific RNA interference (RNAi) construct to silence *HvSec61βa *(AK252927.1), its coding sequence was PCR-amplified using the primer pair Sec61βa_F1 (CACCATGGTGGCTAATGGTGACG) and Sec61βa_R1 (GGGGTGCGGTACAGCTTTC) on cDNA generated from mRNA isolated from 7-day-old barley leaf material. The PCR product was TOPO-cloned into the pENTR/D-TOPO vector (Invitrogen). Positive clones were validated by sequencing. Using Gateway LR cloning, according to the manufacturer’s instructions (Invitrogen), the insert was transferred to the 35S-promoter driven destination vector, pIPKTA30N (Douchkov et al., 2005), to generate the final RNAi construct. To generate the Sec61βa-GFP construct for localization, the full-length Sec61βa coding sequence, without stop-codon, was amplified with the primer pair HvSec61βa_KZK_GWY_FW (GGGGACAAGTTTGTACAAAAAAGCAGGCACCATGGTGGCTAATGGTGACGCCCCT) and HvSec61βa_ns_GWY_Rv (GGGGACCACTTTGTACAAGAAAGCTGGGTTATTAGGGGTGCGGTACAGCTTGCC) on the pENTR clone described above, and using a BP clonase reaction it was cloned into the pDONR201 vector (Invitrogen). Positive clones were confirmed by sequencing. Using a Gateway LR clonase reaction according to the manufacturer’s instructions (Invitrogen), the insert was transferred to the 35S-promoter-driven destination vector, P2GWF7 (Curtis and Grossniklaus, 2003). All final clones were verified by restriction enzyme digestion.

### PARTICLE BOMBARDMENT

Transformation of gene constructs into epidermal cells of 7-day-old barley leaves was conducted by particle bombardment, essentially as described by Douchkov et al. (2005). For transient induced gene silencing (TIGS) studies, the constructs were co-transformed with a β -glucuronidase (GUS) reporter construct, followed by inoculation with *Bgh* 2 days later (inoculation density around 200 conidia per mm^2^). Three days after inoculation, the leaves were GUS-stained, and the relative susceptibility index was calculated by dividing the number of GUS-stained epidermal cells containing a haustorium by the total number of GUS-stained cells. The data were normalized to the empty vector (pIPKTA30N) control. The experiments were repeated at least three times. A cell viability test was performed by co-transformation of the *HvSec61*β*a* RNAi construct or the empty vector control, pIPKTA30N, with the anthocyanin biosynthesis-activating construct, pBC17 (Schweizer et al., 2000). Two days after transformation, the leaves were inoculated with *Bgh* at a density of 200 conidia per mm^2^, and after another 3 days, the anthocyanin-stained cells were counted. Constructs for marker proteins, fused with fluorescent proteins, were transformed and inoculated with *Bgh* 1 day later, and examined by confocal microscopy 2 days after transformation.

### CONFOCAL MICROSCOPY

A Leica SP5-X confocal laser scanning microscope, mounted with a 63 × 1.2 numerical aperture water-immersion objective, was used. For fluorescent protein detection and localization, GFP was excited at 488 nm, and the fluorescence emission was detected between 518 and 540 nm. mCherry fluorescence was excited at 543 nm and fluorescence emission was detected between 590 and 640 nm. 3D projections were created using the Image Surfer 1.2 software^2^ (Feng et al., 2007).

## RESULTS

### *Hv*Sec61βa IS A POTENTIAL SUSCEPTIBILITY FACTOR FOR THE BARLEY POWDERY MILDEW FUNGUS

**FIGURE 1:**
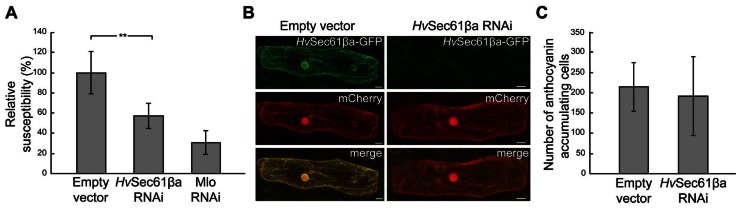
**Silencing of *Hv*Sec61βa reduces susceptibility to *Bgh* without affecting plant cell viability**. **(A)** Susceptibility after co-transformation of empty vector control, *HvSec61*β*a*-RNAi and *Mlo*-RNAi constructs with a GUS-reporter construct into barley epidermal cells, followed by inoculation with *Bgh* 3 days later. The relative susceptibility was calculated as described in Materials and Methods. Mlo-RNAi was used as a positive control. Error bars represent standard deviation (SD). **, *P* < 0.01 (Student’s *t*-test). **(B)** *HvSec61*β*a*-RNAi reduced the GFP signal originating from *Hv*Sec61βa-GFP, but not the fluorescence signal from cytosolic mCherry 5 days after transformation. Micrographs show maximum intensity projections. **(C)** Number of pBC17-transformed cells accumulating anthocyanin, reflecting cell viability, after co-bombardment with either an empty vector control or the *HvSec61*β*a*-RNAi construct on similarly sized pieces of leaf. Two days after bombardment, leaves were inoculated with *Bgh*, and the number of anthocyanin-accumulating cells was scored 3 days later (*n* = 4).

In barley two *Sec61β* genes have been identified, which are named *HvSec61*β*a* and *HvSec61βb*. Interestingly, the *HvSec61*β*a* transcript accumulates in leaves after attack by *Bgh*^3^ (Dash et al., 2012). Therefore, we selected to analyze the role of *HvSec61*β*a* in the barley/*Bgh* interaction, and performed single cell TIGS of this gene. A 35S-promoter-driven RNAi construct, covering the full-length coding region of this gene, was generated and transiently transformed together with a GUS reporter-gene construct into barley epidermal cells (Douchkov et al., 2005). After 2 days, the leaves were inoculated with *Bgh* and transformed cells were stained for GUS activity 3 days thereafter. Infection success of *Bgh* was evaluated microscopically by scoring the total number of GUS-stained cells and the number of GUS-stained cells containing one or more haustoria. Subsequently, the data were normalized to the empty vector control. The RNAi construct of *HvSec61*β*a* resulted in more than 40% reduction in susceptibility to *Bgh *(**Figure 1A**). As a positive control, the relative susceptibility of cells transformed with an *Mlo*-RNAi construct was included (Douchkov et al., 2005). These cells were 70% less susceptible than the control cells. In order to confirm that the RNAi construct in fact results in silencing of *HvSec61*β*a*, we co-transformed barley epidermal cells with the RNAi construct of *HvSec61βa *and a 35S promoter-driven *Hv*Sec61βa-GFP fusion construct. Five days after transformation together with a reference construct for cytosolic mCherry expression, confocal imaging revealed that the RNAi construct prevented appearance of GFP signal, while it did not affect the signal from mCherry in the same cell (**Figure 1B**). The reduced *Hv*Sec61βa-GFP signal indicated that the *HvSec61*β*a* RNAi silencing construct indeed induced degradation of *HvSec61*β*a* encoding mRNA and likely as well impaired endogenous *HvSec61βa *transcript and protein accumulation. Thus, the observed increased resistance of *HvSec61*β*a*-silenced cells indicates a potential role of *Hv*Sec61βa as a susceptibility factor for efficient *Bgh *infection.

In order to analyze whether the reduced susceptibility could be due to reduced viability of the cells in which *HvSec61*β*a* was silenced, a second experiment was performed. Co-transformation was performed with an anthocyanin biosynthesis gene activation construct, pBC17, causing the transformed cells to accumulate the red anthocyanin pigment as long as they stay alive (Schweizer et al., 2000). Two days after transformation, the leaves were inoculated with a high density of *Bgh *conidia (≈200 per mm^2^). Similar numbers of anthocyanin accumulating cells were detected in *HvSec61*β*a*-silenced and non-silenced cells after *Bgh *infection (**Figure 1C**). Therefore, this result confirmed that the *HvSec61*β*a* RNAi construct did not affect the viability of the barley cells after inoculation.

### *Hv*Sec61β LOCALIZATION IN UNINFECTED AND INFECTED BARLEY CELLS

**FIGURE 2:**
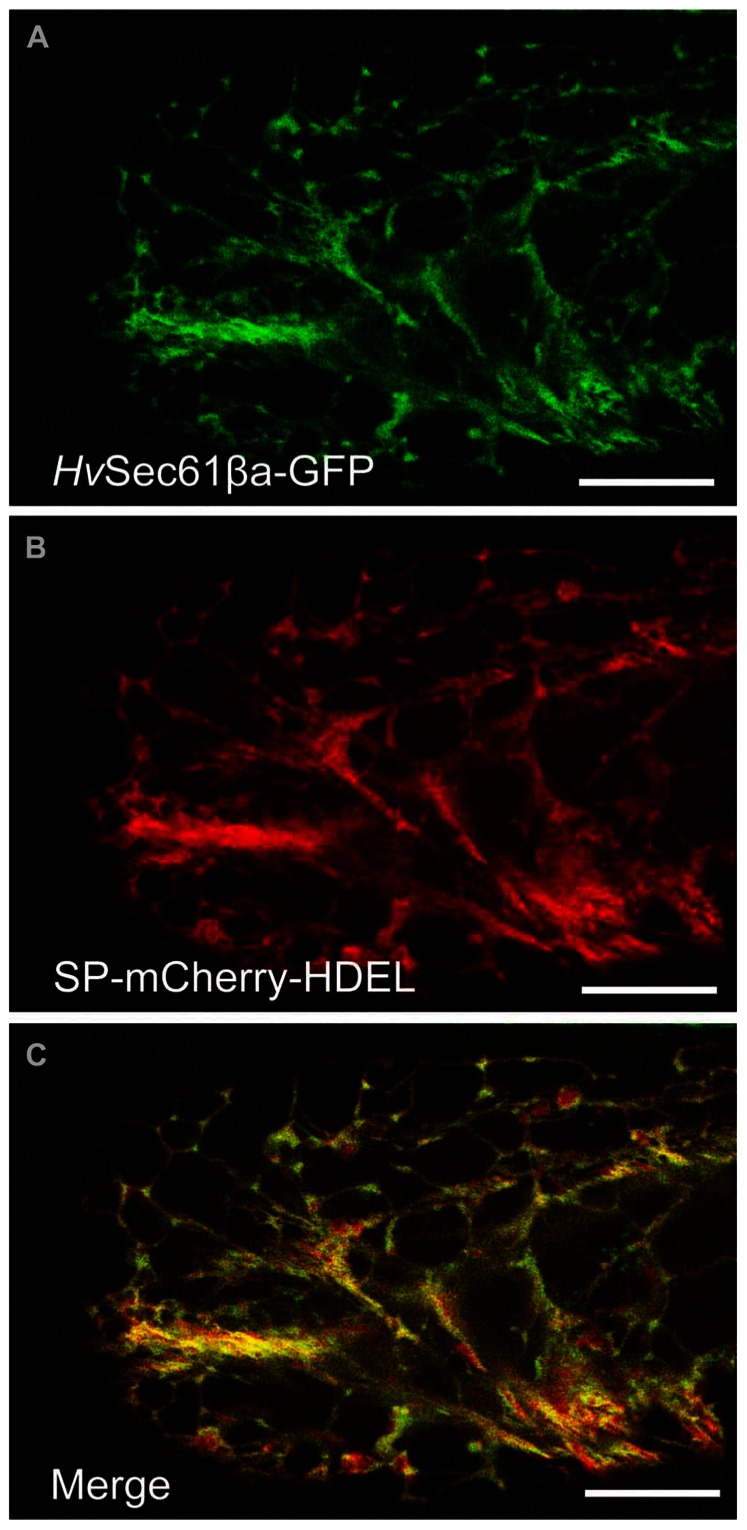
***Hv*Sec61βa co-localizes with an ER luminal marker**. **(A)** Maximum intensity projection of a z-series of confocal images of a barley epidermal cell expressing *Hv*Sec61βa-GFP reveals the ER localization of *Hv*Sec61βa-GFP with the typical distribution within the reticular ER network. **(B)** In the same epidermal cell, the 35S promoter-driven SP-mCherry-HDEL construct is expressed and labels the ER. **(C)** The merged image shows that the *Hv*Sec61βa-GFP and SP-mCherry-HDEL signals largely overlap. Scale bar, 20 μm.

Next we aimed to subcellularly localize *Hv*Sec61βa to search for clues for the powdery mildew-related function of this protein. Sec61β is a small ~8 kDa protein with a single transmembrane domain, and GFP-tagging has previously been used for its localization (Rolls et al., 1999; Voeltz et al., 2006). Therefore, we co-expressed our 35S promoter-driven *Hv*Sec61βa-GFP fusion construct together with a 35S promoter-driven SP-mCherry-HDEL construct (Nelson et al., 2007) in infected and uninfected barley epidermal cells. The SP targets mCherry to the ER and the ER retrieval motif HDEL (His-Asp-Glu-Leu) at the C-terminus retains it in the lumen of the ER (Gomord et al., 1997). Confocal images of epidermal single cells expressing *Hv*Sec61βa-GFP and SP-mCherry-HDEL were recorded 48 h after particle bombardment (**Figure 2**). Intense GFP signal was observed in the ER cortical network throughout the cells expressing GFP-tagged *Hv*Sec61βa (**Figure 2A**). In addition, the *Hv*Sec61βa-GFP signal largely colocalized with mCherry signal from the luminal ER marker (**Figures 2B,C**). The colocalisation is near perfect in the tubular parts of the ER, while the cisternal parts have relatively more mCherry signal. This likely reflects that *Hv*Sec61βa-GFP is membrane bound, and that the soluble mCherry luminal marker dominates the more voluminous cisternal ER. In conclusion, our observations indicate that *Hv*Sec61βa is localized to all parts of the ER.

**FIGURE 3:**
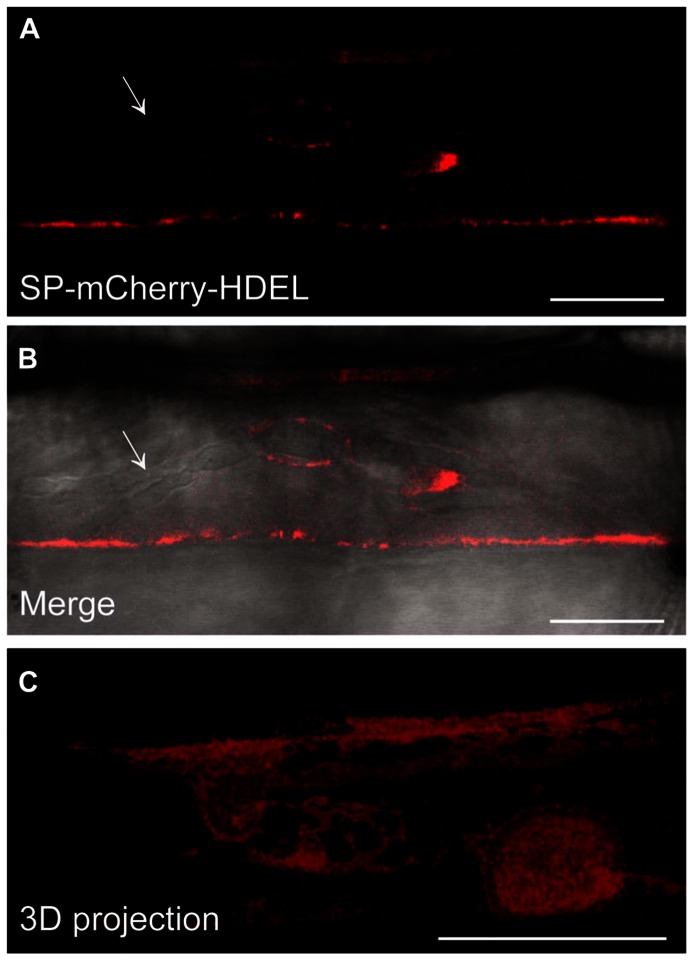
**The SP-mCherry-HDEL ER marker localizes around the *Bgh* haustorial body**. **(A,B)** Confocal images of infected barley epidermal cell 48 h after inoculation with *Bgh*. The fluorescent signal of SP-mCherry-HDEL **(A)** localizes to the ER and surrounds the haustorium inhomogeneously. No fluorescence signal of SP-mCherry-HDEL was observed along the haustorial fingers (arrow). The merged image **(B)** displays the haustorium structure in bright field, overlaid with the fluorescence signal. To visualize the ER tubules around the haustorial body, a 3D projection of a z-series of confocal images **(C)** was generated (Image Surfer 1.2). Scale bar, 10 μm.

Since we confirmed the ER localisation of *Hv*Sec61βa-GFP in barley and have observed increased resistance after silencing this gene, we were interested in knowing how the ER changes its location after pathogen attack. It is often described that infected host cells re-localize organelles and specific proteins, which results in their accumulation at the pathogen attack site (Takemoto et al., 2003; Koh et al., 2005; Caillaud et al., 2012). We used the 35S promoter-driven SP-mCherry-HDEL construct to study the localization of the ER after attack by *Bgh*. Confocal imaging of an infected barley cell revealed that the mCherry ER-luminal marker was located around the body of the *Bgh* haustorium. Meanwhile, this ER marker was most often not present around the haustorial fingers (**Figures 3A,B**). In a 3D projection (**Figure 3C**) of the mCherry fluorescent signal, this distinction between the haustorial body and fingers is clearly visible. These observations revealed that the ER network is in close proximity to the EHM around the haustorial body.

**FIGURE 4:**
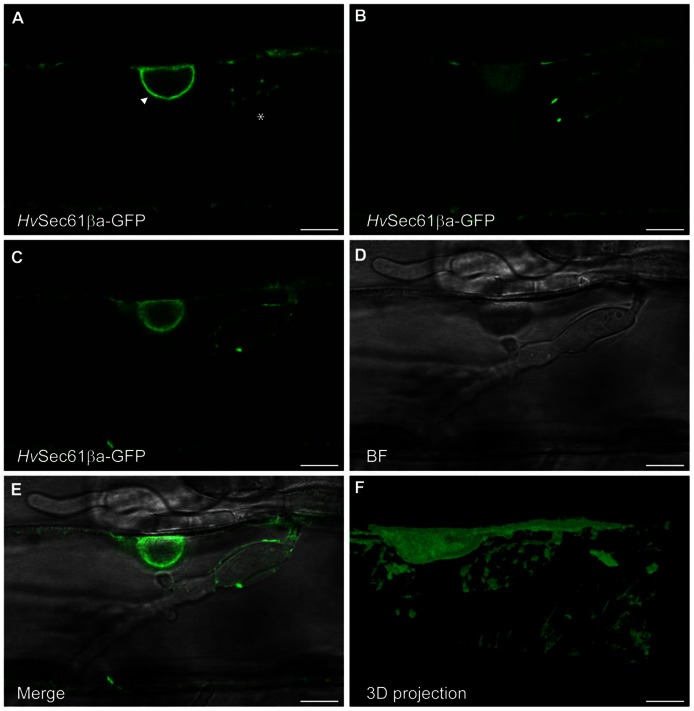
***Hv*Sec61βa-GFP localizes around the *Bgh* haustorial body**. Confocal image of an epidermal cell, transformed with the *Hv*Sec61βa-GFP construct, taken 48 h after *Bgh* inoculation. **(A–C)** Three different focal planes from an image series of an infected cell with a haustorium. *Hv*Sec61βa-GFP localizes to the ER around the nucleus (arrow head, **A**) and surrounds the haustorium in an ER-like tubular pattern (asterisk, **A**). **(C–E)** GFP fluorescence **(C)**, bright field (BF) **(D)** and merged image **(E)** show *Hv*Sec61βa-GFP localization at the surface of the haustorial body. *Hv*Sec61βa-GFP labels the tubular ER network, which is further illustrated in the 3D projection **(F)** (Image Surfer 1.2). Scale bar, 10 μm.

Similar to the mCherry ER-luminal marker (**Figure 3**), the *Hv*Sec61βa-GFP signal was present in the ER network around the *Bgh* haustorial body as well (**Figure 4**). Contiguous accumulation of *Hv*Sec61βa-GFP was detected around the nucleus, which was observed close to the haustorium, supporting the re-localization of this organelle upon pathogen attack (**Figures 4A–E**), as previously described (Schmelzer, 2002). As for the ER-luminal marker, *Hv*Sec61βa-GFP confirmed that the ER and EHM are in close proximity around the haustorial body. In summary, these confocal microscopy results suggest that the *Hv*Sec61βa-GFP-labeled ER is differentially recruited to the proximity of the EHM around the haustorial body, but not around the fingers.

## DISCUSSION

The fact that silencing of *HvSec61*β*a* causes the barley cells to become resistant to powdery mildew suggests that *Hv*Sec61βa either is a negative regulator of defense or a susceptibility factor required for disease. Sec61β is, as described above, associated with protein-transmitting pores in the ER. While it has been barely studied in plants, yeast data suggest that one of its activities is to be part of a post-translational translocon complex, but that this role is not essential under non-stressed conditions (Finke et al., 1996). Furthermore, Sec61β has also been associated with protein retrotranslocation from the ER (Kawaguchi and Ng, 2007; Nakatsukasa and Brodsky, 2008; Willer et al., 2008), and the question is, which of these activities is important in barley cells attacked by *Bgh*.

Silencing of *HvSec61*β*a* would result in inhibition of secretion if this protein is generally required for co- or post-translational protein translocation into the ER. This can hardly explain our phenotype, as inhibition of secretion in barley results in increased susceptibility to *Bgh *(Ostertag et al., 2013). A more likely explanation might be found in a specific *Hv*Sec61βa-function in post-translational translocation. This could involve the so-called “unfolded protein response” (UPR), which results from ER stress due to accumulation of unfolded proteins. During UPR, ER chaperones and components of the ERAD system are up-regulated to prevent the cell from undergoing programmed cell death (Travers et al., 2000). Similarly, ER stress induced by, e.g., tunicamycin (an N-glycosylation inhibitor) increases transcript levels of genes encoding proteins of the ER-QC machinery and the secretory pathway (Martinez and Chrispeels, 2003; Huttner and Strasser, 2012). Recently, a functional link has been established between UPR and pathogen defense in plants. *Arabidopsis* plants mutated in the *IRE1a* gene, encoding a key positive regulator of UPR, were found to have reduced resistance to bacteria (Moreno et al., 2012). An important chaperone that counter acts UPR is the ER- luminal protein, BiP, which is taken up post-translationally through the translocon complex in a Sec61β-dependent manner (Finke et al., 1996). Therefore, a model could be that *HvSec61βa *silencing causes ER-deprivation of BiP, in turn resulting in UPR as well as increased resistance. An *Arabidopsis* *BiP* knock-out line has previously been suggested to be prone for UPR. However, in disagreement with the model, the *BiP* knock-out line had reduced resistance (Wang et al., 2005). This may indirectly suggest that reduced BiP import into the ER is not the cause of the Sec61βa phenotype we observe, while *Bgh* resistance increases in this situation. We therefore favor a function for Sec61βa in protein retrotranslocation in the interaction with the powdery mildew fungus.

**FIGURE 5:**
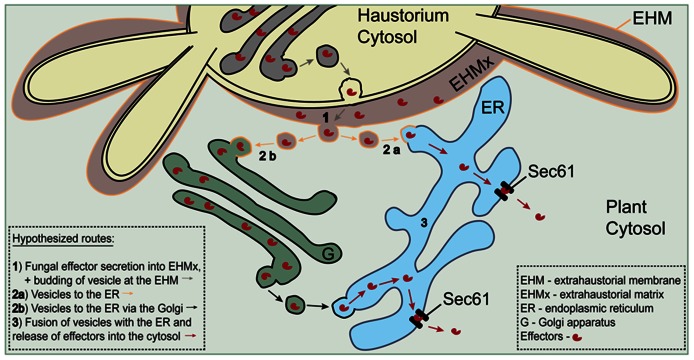
**Schematic model for a possible Sec61-dependent route of effector release into the host cytosol**. Effectors are hypothesized to be transferred from the extrahaustorial matrix to the cytosol through Sec61 retrotranslocon pores in the ER. Trafficking from the matrix to the ER is envisaged to take place in vesicles dependent or independent of Golgi. Adapted from Voegele et al. (2009).

In the meantime, we had an indication of active recruitment of ER by the fungus, supporting that *Hv*Sec61βa functions as a susceptibility component. We observed a close association of the ER, labeled by *Hv*Sec61βa-GFP, and the *Bgh* haustorial body. The ER has also in other cases been found to be closely associated with haustoria (Koh et al., 2005; Micali et al., 2011). However, only *Blumeria* haustoria differentiate in two parts and provide a chance to distinguish variations in ER association. Interestingly, there is little ER association with the haustorial fingers, which could suggest that the ER proximity to the haustorial body is not due to ER being present wherever there is cytosol. Therefore, it is possible that the fungus controls the ER-haustorium association. Voegele et al. (2009) proposed that effector proteins are transferred to the cytosol via the ER. Effectors need to cross a membrane in order to reach the host cytosol, and the ER retrotranslocon pore offers an escape route for this. The resistance phenotype seen after *HvSec61*β*a* silencing is in agreement with a model, where this protein is necessary for pore function. As illustrated in **Figure 5**, we suggest that vesicle trafficking transfers the effectors from the EHMx to the ER in order for them subsequently to employ the retrotranslocon to enter the cytosol. While we consider the model in **Figure 5** to describe the most likely mode of action of Sec61βa in plant powdery mildew interactions, other scenarios are possible. An unexpected function has for instance been described for *Drosophila* Sec61β, which is important for the secretion of the Gurken protein (Kelkar and Dobberstein, 2009). After silencing of *Sec61*β, Gurken left the ER as it normally did in control cells, but subsequently became stalled in the Golgi. Since a control protein still was observed to be secreted after silencing of *Sec61*β, this suggests that Sec61β is required for the Golgi-processing of a subset of the secreted proteins, including Gurken (Kelkar and Dobberstein, 2009). While this cannot be excluded to be due to a retrotranslocon defect, it may indicate that Sec61β also has a function in secretion, which is unrelated to the Sec61 protein pore. Future work will determine which function makes Sec61β important for plant’s susceptibility to the powdery mildew fungus, and whether modification of the gene can be exploited for a disease resistance purpose.

## Conflict of Interest Statement

The authors declare that the research was conducted in the absence of any commercial or financial relationships that could be construed as a potential conflict of interest.

## Footnotes

1 http://webblast.ipk-gatersleben.de/barley/viroblast.php

2 www.imagesurfer.org

3 http://www.plexdb.org/plex.php?database=Barley
